# The Care of Children's Teeth

**Published:** 1895-12

**Authors:** 


### Editorial, Etc.

THE CARE OF CHILDREN'S TEETH.—In the August (1892) nnmber of this Journal we said—under the head

THE DENTIST A TEACHER.

It is admitted that children's teeth do not receive the degree of attention which they demand. The public mind believes that because the period of duration of the temporary teeth is a brief one, they are not worthy the care which is bestowed upon the permanent set. Children are given little, if any, dental treatment until they are about ten years old, by which time, through the ignorance or neglect of parents almost irreparable damage has been done to the sixth year molars, large teeth well adapted for mastication. Physicians and dentists by statistics have shown that 56 per cent. of the upper molars are decayed before the seventh year, and that the lower molars, coming first, decay at once; 20 per cent. during the first year, and 70 per cent. bythe third year, after eruption.

The number of teeth lost is now so extensive that it assumes a national calamity, for the digestion bəing impaired the physical stamia of the nation is injured. The evils arising from insufficient mastication caused by the loss of teeth can not be exaggerated. The dental profession is, in a great measure, responsible for the ignorance respecting the mouth and the teeth. If schools would engage the services of properly-qualified dentists, the needless loss of many teeth may be prevented. Instruction in the daily care of the teeth could be given.

The subject is now receiving special attention. The American Dental Association at its meeting in Asbury Park, in August, "regards the suggestion as to the education of children in the proper care of their teeth as of great interest and suggests further discussion upon it," the Board of Dental Examiners of Tennessee at the request of the State Dental Association had printed and distributed throughout the state copies of the following.

To Instructors in the Public Schools in the State of Tennessee:

RECOGNIZING the necessity of impressing upon children early the importance of the care and preservation of their teeth, the Tennessee Dental Association, at its meeting in Knoxville in August last, requested us to prepare an essay inviting your attention to this vital subject. You are requested to impress upon your pupils that it is of greater consequence to their health and happiness to observe the few rules here laid down than the observance of most other hygienic laws.

Placed at the beginning of the alimentary canal, our teeth hold an important relation to the digestive function, and through it to the entire body. They are inseparably connected with the nervous, circulatory, and respiratory systems. Henee their preservation constitutes an important art in medicine; for without healthy teeth, there cannot be thorough mastication; without thorough mastication, there cannot be perfect digestion; without perfect digestion, there cannot be proper assimilation; without proper assimilation there cannot be nutrition; without nutrition, there cannot be health; without health, it is impossible to attain the highest perfection of mental, moral, and physical growth.

Upon the soundness and perfection of the teeth, therefore, much depends as regards the health, comfort, personal appearance, and voice. Every one should recognize the importance of their preservation, and impress upon children early in life the necessity of keeping their teeth and mouths clean and in good hygienic condition; for if neglected, the teeth frequently become not only diseased and produce great suffering, but are lost before middle life.

Decay is not the only disease to be combated, but frequent irregularities, incrustations of tartar, diseases of the gums, and around their necks, producing foul breaths, disordered stomachs, and indigestion.

Children should be advised to rub their gums with soft cloths and thoroughly brush their teeth, freeing all spaces between them from food and other accumulations, after each meal and before retiring.

Whenever cavities of decay exist they should be stopped, to prevent the further destruction of the tooth and avoid the loss of its function.

That the children of the State of Tennessee may be impressed with these facts, we address this notice to you, begging that you will not only now, but through the coming sessions, repeatedly endeavor to bring to their attention the important truths set forth in this circular, and thus help the dental profession in its efforts to add to the comfort, happiness, and average length of human life.

Lady Aberdeen, wife of the Governor-General of Canada, has made a suggestion, says the Buffalo Courier, which may lead to action on the part of the overseers of public instruction in the Dominion. The suggestion, continues the Courier, is that the teeth of the pupils in the public schools should be regularly inspected by experts appointed by the government. Inasmuch as none of our organs is so much injured by neglect as the teeth, and children try to avoid caring for them because of the bother, the plan which Lady Aberdeen suggests has some merit. The general vaccination of the children in the public schools is conceded to be necessary, but as a matt er of fact the public health and public good looks are in just about as much peril from bad teeti as from small-pox. How much more pleasing to the eye would the next generation be if expert inspectors would show in the public schools the benefits arising from a care of the teeth! Many a beautiful face which nowadays when it breaks into a smile, at once becomes disappointing would then be rendered only more attractive by the twin rows of white, firm teeth shining between the parted lips. It is to be hoped that Canada will make some experiments in this matter and report upon the result of them.

The Youth's Companion has this to say about good teeth in children:

"All diseases which profoundly affect the nutrition influence the development of the teeth; and since the growth of the teeth is mainly limited to the age of childhood, their condition is especially influenced by children's diseases.

"Faulty nutrition or severe wasting illness show themselves nowhere more prominently than in the development of the bones and teeth; and on the other hand good teeth in children play a very important part in producing a healthful and robust manhood or womanhood. Decaying or loosened teeth directly favor imperfect mastication and consequent indigestion.

"Indigestion favors poor nutrition; it causes the secretions of the mouth to become acid in reaction—a perversion of the normal reaction of the saliva, which attacks the teeth and favors their rapid decay.

"A case of this kind has lately been observed. A child, naturally good-natured but "spoiled" by indulgent parents, was allowed to eat fresh cakes, expensive candies, jams, pastries. Attacks of indigestion, with vomiting of the acid contents of the stomach, supervened. The teeth softened, "became poor" at an early age.

"In order to assure such a child a healthy bodily development and protect it from the evils of subsequent attacks of indigestion, there must be something more than a correction of its diet. The teeth should be filled. This guards against disease of the alveolar process, or the bony portion of the jaw into which the roots of the teeth are inserted, against an involvement of the second tooth, just developing beneath the first.

"A regular supervision of children's teeth would save large dentist's bills, and would undoubtedly tend to a healthier, stronger race of mankind. From the time of the first appearance of the teeth through the gums, they should be subjected to a rubbing twice a day with a soft rag and lime water, until the twelfth month of infancy, when a soft brush should be substituted.

"Frequent visits to the dentist are an absolute necessity.

"Children who are allowed to eat warm bread, rich pastries, cakes and candies are almost invariably subject to habitual attacks of indigestion. The far-reaching effects of such attacks can be avoided by the prohibition of such food. Meat, not too tender, and crusts of bread are excellent objects upon which a child's teeth may be exercised and strengthened."

And Harper's Weekly concludes an article with these words: "Take it all in all, care of the teeth pays in comfort, in beauty, in the conservation of health from youth to old age." R. G.

---

